# Mitochondrial BNIP3 upregulation precedes endonuclease G translocation in hippocampal neuronal death following oxygen-glucose deprivation

**DOI:** 10.1186/1471-2202-10-113

**Published:** 2009-09-08

**Authors:** Shen-Ting Zhao, Ming Chen, Shu-Ji Li, Ming-Hai Zhang, Bo-Xing Li, Manas Das, Jonathan C Bean, Ji-Ming Kong, Xin-Hong Zhu, Tian-Ming Gao

**Affiliations:** 1 Department of Neurobiology, Southern Medical University, Guangzhou, PR China; 2 Department of Human Anatomy and Cell Science, University of Manitoba Faculty of Medicine, Winnipeg, Man., Canada; 3 Department of Neuroscience, Institute of Molecular Medicine and Genetics, Medical College of Georgia, Augusta, Georgia, USA

## Abstract

**Background:**

Caspase-independent apoptotic pathways are suggested as a mechanism for the delayed neuronal death following ischemic insult. However, the underlying signalling mechanisms are largely unknown. Recent studies imply the involvement of several mitochondrial proteins, including endonuclease G (EndoG) and Bcl-2/adenovirus E1B 19 kDa-interacting protein (BNIP3), in the pathway of non-neuronal cells.

**Results:**

In this report, using western blot analysis and immunocytochemistry, we found that EndoG upregulates and translocates from mitochondria to nucleus in a time-dependent manner in cultured hippocampal neurons following oxygen-glucose deprivation (OGD). Moreover, the translocation of EndoG occurs hours before the observable nuclear pyknosis. Importantly, the mitochondrial upregulation of BNIP3 precedes the translocation of EndoG. Forced expression of BNIP3 increases the nuclear translocation of EndoG and neuronal death while knockdown of BNIP3 decreases the OGD-induced nuclear translocation of EndoG and neuronal death.

**Conclusion:**

These results suggest that BNIP3 and EndoG play important roles in hippocampal neuronal apoptosis following ischemia, and mitochondrial BNIP3 is a signal protein upstream of EndoG that can induce neuronal death.

## Background

The hippocampus, a very important structure for learning and memory, is among the most vulnerable brain regions after global cerebral ischemia. The rapid decrease of oxygen and glucose in the ischemic region can trigger delayed neuronal death [1], and reperfusion may further exacerbate the injury. The delayed cell death occurs primarily through an apoptotic mechanism [2]. Evidence has accumulated that a large proportion of the delayed neuronal death is mediated by caspase-independent pathways [3-5]. However, the signalling mechanisms remain largely unclear. Recent studies have implicated mitochondrial proteins, such as endonuclease G (EndoG) and Bcl-2/adenovirus E1B 19 kDa-interacting protein (BNIP3), as players involved in this pathway in non-neuronal cells [6].

EndoG is an endonuclease normally localized in the mitochondrial intermembrane space, and translocates to the nucleus when cells are exposed to an apoptosis-inducing stimulus. After moving to the nucleus, EndoG cleaves chromatin DNA into nucleosomal fragments independent of caspases [7]. The compartmentalization of mitochondria plays the major role in EndoG trafficking. The EndoG location site might indicate that this enzyme is not an instrument for immediate response to cell injury [8].

BNIP3 is a member of a unique family of death-inducing mitochondrial proteins [9]. In neurons, the expression of BNIP3 is undetectable under normal conditions [10] but can be induced by hypoxia/ischemia and oxidative stress [11,12]. The BNIP3-induced non-neuronal cell death is characterized by mitochondrial damage but is independent of caspase activation and cytochrome c release [10]. It is presently unclear how BNIP3 initiates neuronal death.

In this study, we profiled BNIP3 and EndoG expression and translocation in cultured hippocampal neurons following oxygen-glucose deprivation (OGD). We demonstrate here that EndoG upregulates and translocates from mitochondria to nucleus in a time-dependent manner. Moreover, the translocation of EndoG occurs hours before the observable nuclear pyknosis. Importantly, we investigated the causal relationship between mitochondrial BNIP3 upregulation and EndoG translocation and nuclear pyknosis by over expressing or knocking down of BNIP3. Our findings strongly support a role for BNIP3 as a signal protein upstream of EndoG leading to the induction of neuronal death.

## Results

### OGD induces EndoG upregulation in primary neuronal cultures

**Figure 1:**
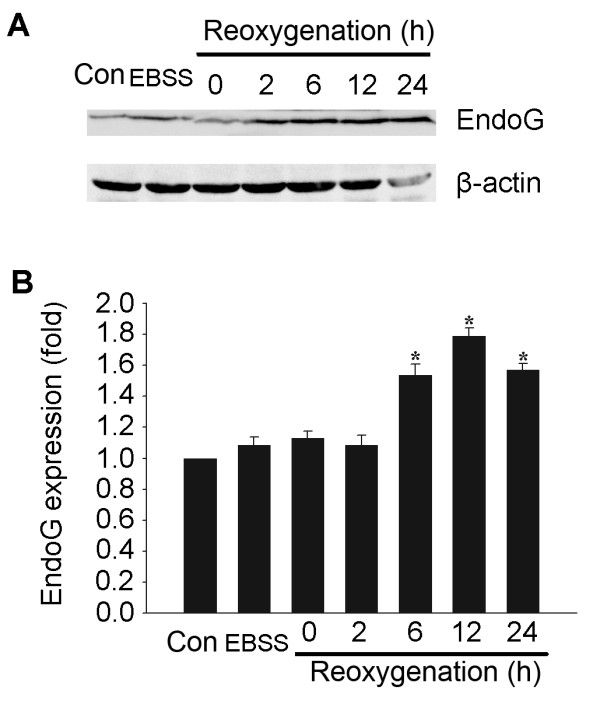
**Upregulation of EndoG expression in reoxygenated hippocampal neuronal cultures following OGD**. (A) Representative western blot showing the expression of EndoG. (B) Quantification of the expression of EndoG. Data were presented as fold of the control group. *p < 0.05 vs. control group, n = 6.

Primary rat hippocampal neurons at day 12 in vitro were subjected to OGD for 4 h and reoxygenation for 0 h, 2 h, 6 h, 12 h and 24 h. Normal cultured control and EBSS-treated neurons under normoxic condition were set as controls. Levels of EndoG expression were determined by western blot analysis. EndoG immunoreactivity was identified as the ~35 kDa protein band in the whole cell fraction after treatment. Immunoblotting with a monoclonal β-actin antibody was performed as standards for calculation of EndoG expression. As shown in Figure 1, total amount of EndoG did not vary until reoxygenation was prolonged to 6 h and accumulated to the highest level at 12 h of reoxygenation.

### EndoG translocates from the mitochondria to the nucleus after reoxygenation

**Figure 2:**
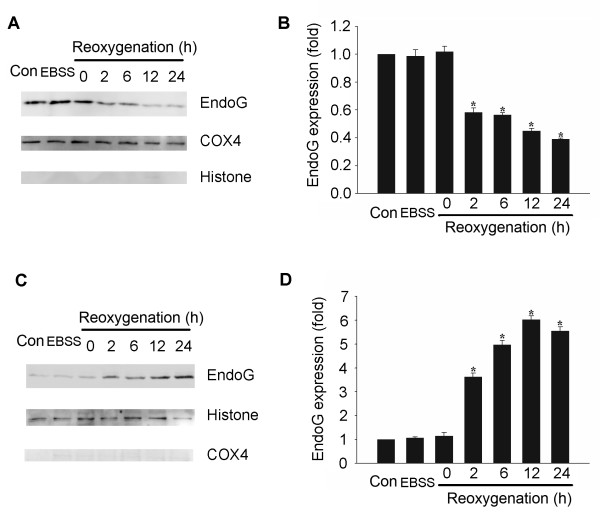
**Translocation of EndoG from mitochondria to nuclei following reoxygenation**. (A) Representative western blot showing the level of mitochondrial EndoG. (B) Quantification of the level of mitochondrial EndoG. (C) Representative Western blot showing the level of nuclear EndoG. (D) Quantification of the level of nuclear EndoG. Data were presented as fold of the control group. *p < 0.05 vs. control group, n = 6.

We next examined the subcellular localization of EndoG to determine whether OGD could induce the translocation of EndoG. Mitochondrial and nuclear fractions were prepared by differential centrifugation. Cox IV antibody for mitochondrial and histone H3 antibody for nuclear fractions were used as sample loading controls. EndoG level in the mitochondrial fraction began to decrease significantly at 2 h of reoxygenation and further decreased with time exposed to reoxygenation (Figure 2A and 2B). Meanwhile, EndoG in nuclear fraction showed a significant increase at the corresponding time point and accumulated with increased periods of reoxygenation (Figure 2C and 2D). These data suggest that the released EndoG from mitochondria translocates to nucleus.

### Nuclear EndoG translocation precedes OGD-induced neuronal death

**Figure 3:**
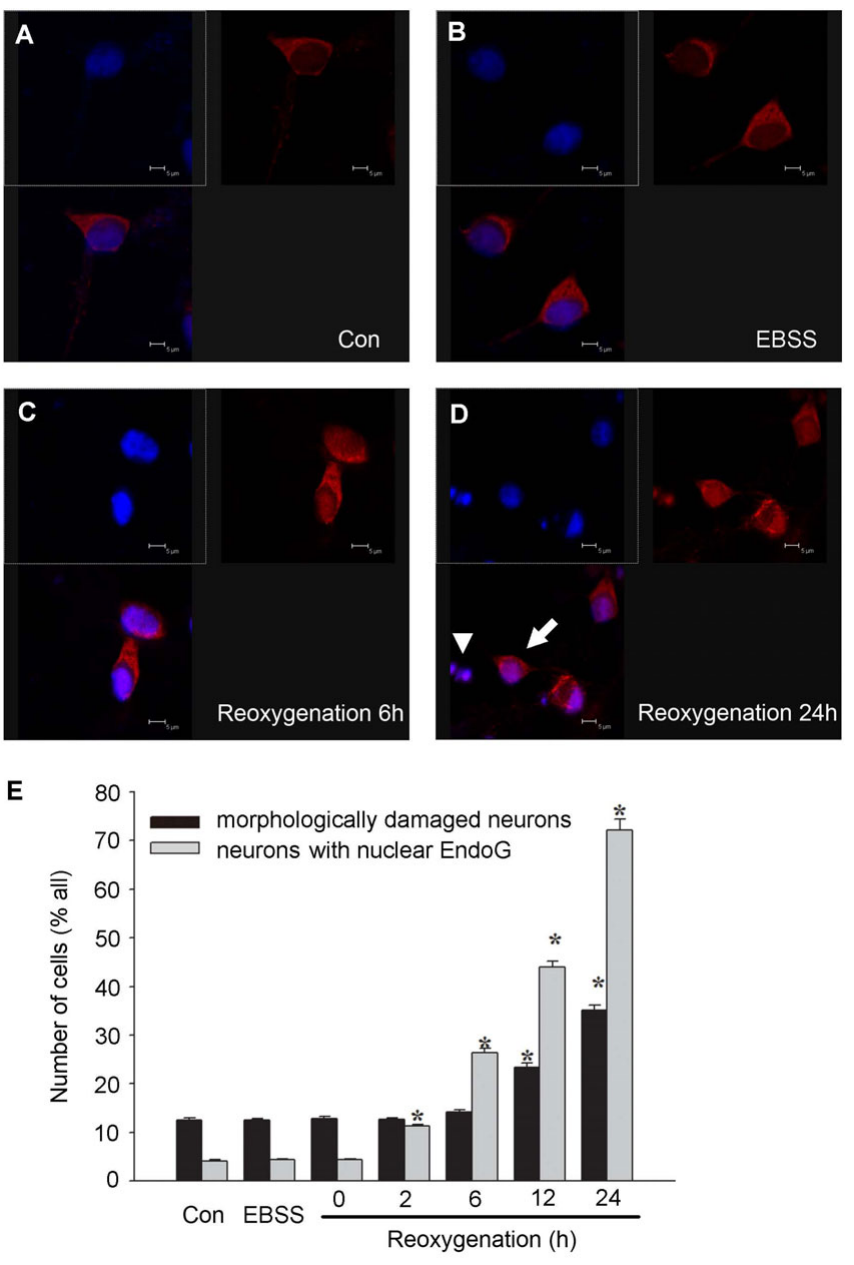
**EndoG nuclear translocation preceded neuronal cell death following reoxygenation**. (A-D) Confocal laser scanning microscope images of EndoG immunoreactivity (red) at control, EBSS, OGD/R6 h, OGD/R24 h, respectively. Co-staining with DAPI (blue) allowed the identification of nuclear translocation of EndoG. The white arrow indicates the nucleus with EndoG translocation but without pyknosis, and the white arrowhead indicates the pyknotic nucleus with EndoG translocation; (E) Percentage of damaged neurons and neurons displaying nuclear EndoG at different time points following reoxygenation. *p < 0.05 vs. control group, n = 9.

To evaluate the role of nuclear EndoG translocation in OGD, we investigated the relation between EndoG translocation and cell death. At different time points after reoxygenation, the intracellular localization of EndoG was detected by immunocytochemistry; the morphologically damaged neurons were identified by condensed and fragmented nuclei using DAPI nuclear staining. The result revealed that EndoG was mainly located in the intracellular space out of the nucleus shown as red fluorescence in normal control group or in EBSS-treated group under normoxic condition (Figure 3A and 3B). Consistent with the results from the previous western blot analysis, the nuclear translocation of EndoG started at 2 h after reoxygenation and increased progressively (Figure 3C-E). Significant numbers of neurons displaying EndoG nuclear staining were observed 6 h after reoxygenation, a time point when no obvious morphological neuronal damage could be observed (Figure 3C and 3E). Thereafter, although the number of morphologically damaged neurons also increased with time, a great quantity of pyknotic nuclei could not be observed until 12 h after reoxygenation. Furthermore, the proportions of morphologically damaged neurons were less than that of the EndoG translocated neurons at 12 h and 24 h, respectively (Figure 3D and 3E). These results demonstrate that EndoG translocation from mitochondria to nucleus precedes neuronal cell death.

### BNIP3 mediates OGD-induced hippocampal neuronal death

**Figure 4:**
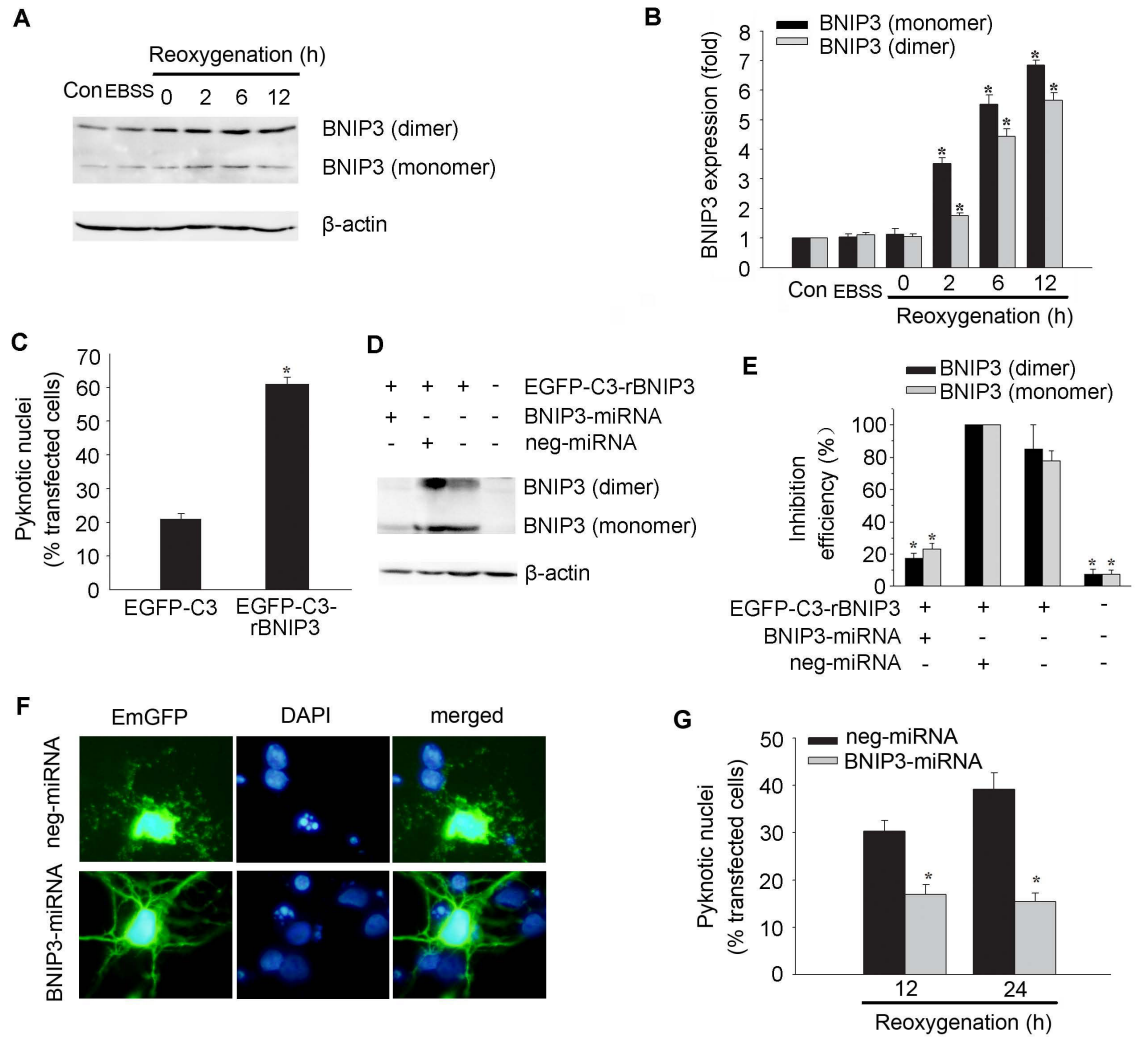
**BNIP3 is a mediator of OGD-induced hippocampal neuronal death**. (A) Representative western blot showing the expression of BNIP3. (B) Quantification of the expression of BNIP3. Data were presented as fold of the control group. *p < 0.05 vs. control group, n = 3. (C) Forced expression of BNIP3 by transfection with EGFP-C3-rBNIP3 plasmid in normal cultured hippocampal neurons induced cell death. EGFP-C3 plasmid was transfected to neurons as a control. *p < 0.05, n = 21-29. (D-E) Inhibition efficiency of BNIP3-miRNA on BNIP3 expression in HEK293 cells co-transfected with indicated plasmids. *p < 0.05 vs. neg-miRNA transfected control, n = 3. (F) Cultured neurons were transfected with BNIP3-miRNA or neg-miRNA 48 h before OGD. Cell death of positive transfected neurons (green) was assessed by nuclear DAPI staining (blue) after OGD. Neurons pretreated with BNIP3-miRNA showed less pyknosis 12 h after reoxygenation compared with neg-miRNA treated neurons. (G) Quantification of experiments described in (F). Pretreatment with BNIP3-miRNA significantly reduced cell death compared with neg-miRNA treated controls. *p < 0.05 vs. control group, n = 21-26.

To explore the role of BNIP3 in OGD-induced hippocampal neuronal cell death, we first observed the expression of BNIP3 following reoxygenation. Western blot analysis revealed that BNIP3 bands with molecular weights of 30 kD (monomer) and 60 kD (dimer) started to increase at 2 h of reoxygenation (Figure 4A and 4B). We next examined whether the expression of BNIP3 was sufficient to induce neuronal death and also if BNIP3 was necessary to mediate neuronal death after OGD. Hippocampal neurons were transfected with pEGFP-C3-rBNIP3 plasmid or pEGFP-C3 plasmid as a control. As shown in Figure 4C, exogenous BNIP3 expression in normal cultured neurons led to about 40% of cell death after transfection for 24 h. To knockdown the expression of BNIP3, BNIP3-miRNA construct was generated and co-transfected with pEGFP-C3-rBNIP3 plasmid encoding the full length of rat BNIP3 in HEK293 cells. The inhibition efficiency of BNIP3-miRNA for BNIP3 expression was 80% compared with the neg-miRNA transfected controls as identified by western blot analysis (Figure 4D and 4E). Addition of BNIP3-miRNA to hippocampal neurons significantly reduced OGD-induced nuclear pyknosis when detected 12 and 24 h after reoxygenation (Figure 4F and 4G).

### BNIP3 initiates the EndoG translocation

**Figure 5:**
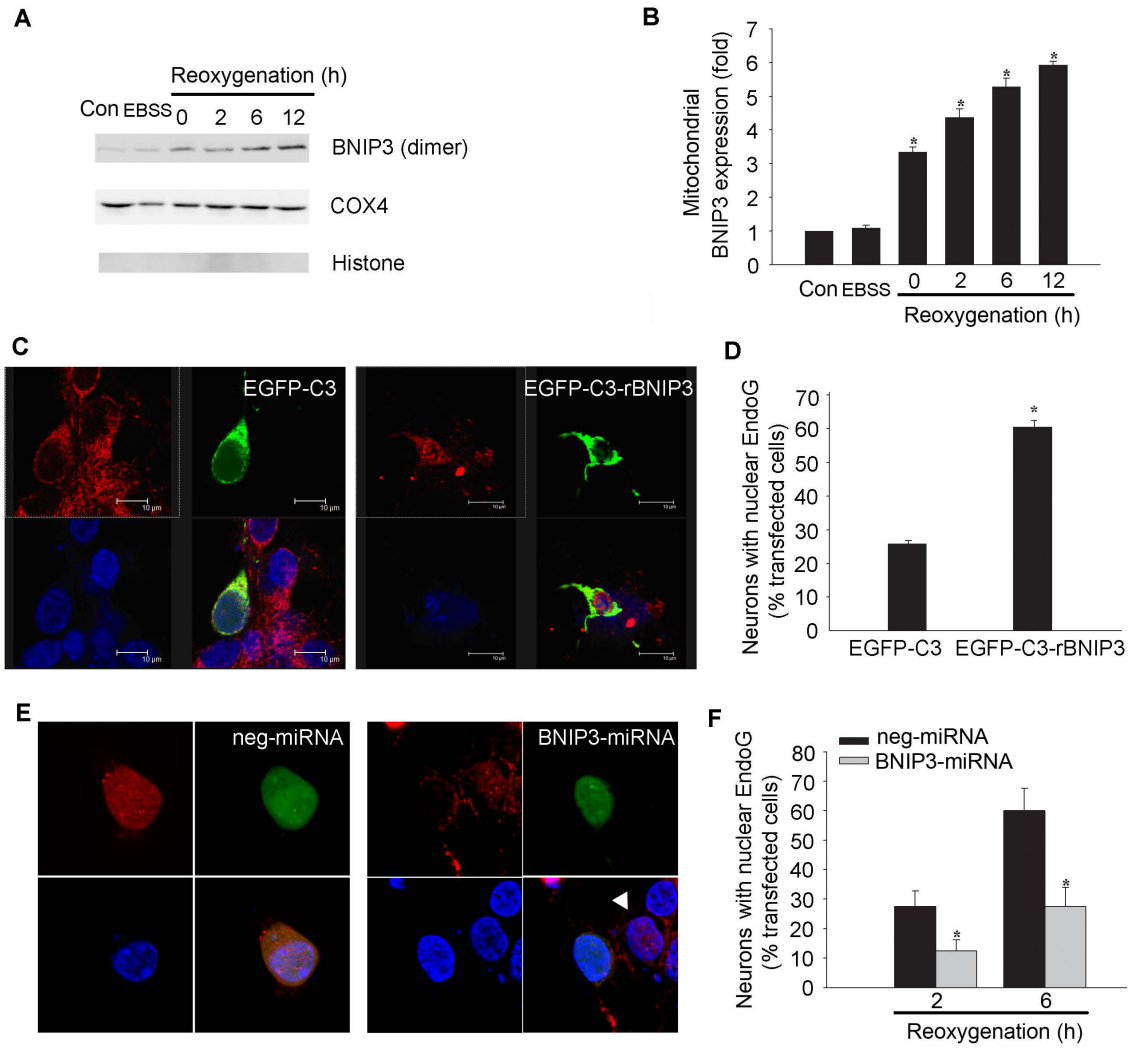
**BNIP3 initiates the nuclear translocation of EndoG following reoxygenation**. (A) Representative western blot showing the expression of mitochondrial BNIP3. (B) Quantification of the expression of mitochondrial BNIP3. Data were presented as fold of the control group. *p < 0.05 vs. control group, n = 3. (C) EndoG (red) is located outside the DAPI-stained nucleus (blue) in EGFP-C3 transfected hippocampal neurons (left panel). EndoG translocated to the DAPI-stained nucleus in EGFP-C3-rBNIP3 transfected hippocampal neurons (right panel). (D) Percentage of neurons displaying nuclear EndoG in EGFP-C3 and EGFP-C3-rBNIP3 transfected hippocampal neurons, respectively. Data was presented as the percentage of the transfected cells. *p < 0.05 vs. control group, n = 21-26. (E) EndoG translocated to the nucleus in neg-miRNA transfected hippocampal neurons (left panel) at 6 h after reoxygenation. EndoG located outside of the nucleus in BNIP3-miRNA transfected neurons (right panel) at 6 h after reoxygenation. Arrowhead showed that the EndoG still translocated to the nucleus in BNIP3-miRNA untransfected neurons (right panel) at 6 h after reoxygenation. (F) Percentage of neurons displaying nuclear EndoG in neg-miRNA and BNIP3-miRNA transfected hippocampal neurons, respectively. Data are presented as the percentage of the positive transfected cells. *p < 0.05 vs. control group, n = 40-50.

Finally, we tested the hypothesis that BNIP3 initiates the translocation of EndoG to nucleus in OGD-induced neuronal death. Because BNIP3 predominantly localizes to the mitochondria and homodimerization appears to be a feature of mitochondrial localization and important for BNIP3's function [9], we explored the subcellular localization of OGD-induced BNIP3. Immediately after reoxygenation, upregulation of mitochondrial BNIP3 (dimer) was detected, 2 h earlier than the EndoG translocation (Figure 5A and 5B). We next examined whether forced expression of BNIP3 induced EndoG translocation. As shown in Figure 5C and 5D, the proportion of nuclear EndoG positive staining neurons increased significantly in exogenous BNIP3 expressing neurons compared to that in pEGFP-C3 transfected control neurons. Furthermore, we explored whether knockdown of BNIP3 could reduce the OGD-induced nuclear translocation of EndoG. Based on the earlier result that EndoG began to significantly translocate to nucleus at 2 h after reoxygenation, we wanted to observe the effect that BNIP3 inhibition would have on the percentage of neurons with nuclear EndoG at 2 h and 6 h after reoxygenation, respectively. Knockdown of BNIP3 expression significantly reduced EndoG nuclear translocation (Figure 5E and 5F).

## Discussion

Recent biochemical and genetic studies have revealed that EndoG is an important mitochondrial protein that emanates from the mitochondria during apoptosis and facilitates degradation of nuclear chromatin [7,13]. The present study, for the first time, strongly implies an essential role of EndoG in post-ischemic hippocampal neuronal cell death in vitro. Western blot analysis and immunocytochemistry demonstrate that EndoG not only upregulates in hippocampal neurons following reoxygenation but also translocates from mitochondria to nucleus. This finding is consistent with previous results on other ischemic models, including cortical neuronal cultures subjected to hypoxia [11], and cortical neurons of mice subjected to transient or permanent focal cerebral ischemia [14,15]. Using subcellular fractionation with a detailed time course, we confirmed that EndoG translocation markedly precedes the appearance of biochemical markers of cell death. The good temporal and spatial relationship between EndoG translocation and nuclear pyknosis suggests a causal role of EndoG translocation in OGD-induced hippocampal neuronal death.

The present study also identifies BNIP3 and EndoG translocation as early events in hippocampal neurons subjected to OGD. The increase in mitochondrial BNIP3 expression was observed immediately after reoxygenation. The translocation of EndoG was detectable at 2 h and increased significantly at 6 h after reoxygenation. This is consistent with a previous report in a mouse model of brain ischemia, in which EndoG translocation was observed 4 h after transient focal cerebral ischemia [14]. Although in a previous study we found that BNIP3 and EndoG translocation occurred relatively late in cultured cortical neurons subjected to hypoxia [11], the mitochondrial translocation of the BNIP3 was before the nuclear translocation of the EndoG in both studies, which strongly supports that BNIP3 acts as an upstream signal of EndoG. The discrepancy might result from either different neuron types examined or different ischemic models used. Compared with hypoxia, OGD is a more severe stress to neurons and re-supply of oxygen and glucose may further exacerbate the injury. In fact, we detected an upregulation of BNIP3 in mitochondria at 0 h after reoxygenation, which might imply that the mitochondrial translocation of BNIP3 occurred and the signalling cascade triggered by BNIP3 started during OGD. The expression of BNIP3 in mitochondria further increased with prolonged reoxygenation, and knockdown of BNIP3 reduced cell death, supporting a role of BNIP3 in activating the cell death program. We also found that forced expression of BNIP3 increased nuclear translocation of EndoG. On the other hand, knockdown of BNIP3 expression reduced OGD-induced EndoG translocation. Therefore, the present findings argue for the importance of mitochondrial BNIP3 upregulation as an upstream modulator of EndoG translocation in ischemic neuronal injury.

## Conclusion

In the present study we provide the first detailed description of the time-dependent subcellular localization of EndoG and BNIP3 in the cultured hippocampal neurons following OGD. By exploring the temporal relationship between the neuronal nuclear pyknosis and the translocation of the mitochondrial death-related proteins, BNIP3 and EndoG, we have been able to suggest an important role of mitochondrial BNIP3 upregulation and EndoG translocation in neuronal death. Combined with the findings that forced expression of BNIP3 increases EndoG translocation and neuronal death, and knockdown of BNIP3 decreases EndoG translocation and neuronal death, our results support the role of mitochondrial BNIP3 as a signal protein upstream of EndoG leading to the induction of neuronal death.

## Methods

### Animals

Neonatal 1 d Sprague-Dawley rats were provided by Southern Medical University and conditions regarding health and hygiene were confirmed. All experimental procedures in this study were performed within National Institutes of Health guidelines for the care and use of laboratory animals.

### Cell culture and OGD

HEK293 cells were cultured in DMEM (Invitrogen) supplemented with 10% heat-inactivated fetal calf serum and 2 mM glutamine. Primary hippocampal neuronal cultures were prepared from neonatal Sprague-Dawley rats (P1) as described previously [16]. Cells were cultured in Neurobasal medium supplemented with 2% (v/v) B27, 2 mM glutamine, and 100 U/ml penicillin/streptomycin (Invitrogen, San Diego, CA). Cultures contain > 95% neurons as routinely controlled by neuronal-specific nuclear protein (NeuN) immunostaining. In 12- to 13-day-old primary neurons, the control culture medium was replaced with Earl's balanced salt solution (EBSS) medium (in mg/L: 6800 NaCl, 400 KCl, 264 CaCl_2_·2H_2_O, 200 MgCl_2_·7H_2_O, 2200 NaHCO_3_, 140 NaH_2_PO4·H_2_O, and 1000 glucose, pH 7.2). For OGD, glucose-free EBSS medium was purged with N_2_/CO_2_/O_2 _(94%/5%/1%) for 30 min, resulting in an oxygen content of 2-3%. Neurons were then washed three times with this medium and incubated for 4 h in an oxygen-free N_2_/CO_2_/O_2 _(94%/5%/1%) atmosphere. Thereafter, the medium was replaced by standard culture medium (see above). Cells were collected at 0, 2, 6, 12 and 24 h after reoxygenation respectively for western blot analysis, immunocytochemistry and quantification of cell death.

### Preparation of the mitochondrial and nuclear fractions

Mitochondrial protein was extracted according to the manufacturer's protocol (PIERCE). Nuclear fractions were prepared as described by Yu et al. with slight modifications [17]. Briefly, the cells were washed and resuspended in isotonic homogenization buffer (250 mM sucrose, 10 mM KCl, 1.5 mM MgCl_2_, 1 mM Na-EDTA, 1 mM Na-EGTA, 1 mM dithiothreitol, 0.1 mM phenylmethylsulfonylfluoride, 10 mM Tris-HCl, pH 7.4) containing a proteinase inhibitor cocktail (4-(2-aminoethyl)benzenesulfonyl fluoride (AEBSF), pepstatinA, E-64, bestatin, leupeptin, and aprotinin, Sigma). After homogenization, the homogenate was trypsinized with 0.1% trypsin for 5 min, and the unbroken cells were spun down. Then, the nuclei were fractionated at 80 × g for 10 min from the supernatant and washed three times with homogenization buffer containing 0.01% NP-40.

### Western Blot analysis

Western blot analysis was performed as previously described [18]. Protein samples (30 μg protein) were separated on 12% polyacrylamide gels and transferred to polyvinylidene diflouride membrane. The membrane was incubated with 5% skimmed milk in Tris-buffered saline for 1 h to block nonspecific binding. The membrane was then incubated overnight at 4°C with anti-EndoG antibody (1:500; Abcam), anti-BNIP3 (1:500; Sigma), anti-COX IV (1:5000; Abcam) or anti-Histone H3 (1:5000; BOSTER, Wuhan, China) and then further incubated with horseradish peroxidase-conjugated secondary antibodies (1:5000) for 1 h. Immunoblotting was detected by ECL (Key GEN, Nanjing, China) and imaged on a FluorChem 8900 imager (Alpha Innotech). Western blot bands were quantified using the FluorChem™ SP V.5.0.2.4 software.

### Immunocytochemistry

Cultured cells were fixed with 4% formaldehyde in PBS for 15 min and nonspecific binding was blocked for 60 min in PBS containing 0.3% Triton X-100 and 3% BSA. Anti-EndoG antibody (1:50) was applied in PBS containing 0.3% Triton X-100, incubated overnight at 4°C, followed by a Rhodamine (TRITC)-conjugated AffiniPure Goat Anti-Rabbit IgG (H+L) (Jackson) secondary antibody (1:200) for 60 min. Negative controls, in which the primary antibody was omitted, were similarly treated as described above. Nuclear staining was followed with the fluorescent DNA-binding dye 4', 6-diamidino-2-phenylindole dihydrochloride (DAPI) (1:1000; Roche) for 15 min. Fluorescence pictures were taken on a Leica TCS SP2 scanning confocal microscope. Neurons with condensed and fragmented nuclei were considered apoptotic. Five randomly chosen visual fields were counted in each sample by an investigator blind to the experimental conditions. About 500 cells from each group were counted.

### BNIP3 gene silencing

Interfering plasmids targeting BNIP3 (BNIP3-miRNA) were generated using BLOCK-iT Pol II miR RNAi Expression Vector Kits (Invitrogen) following the instructions of the manufacturer. The miRNA sequences were designed using Invitrogen's BLOCT-iT RNAi Designer. Briefly, the oligonucleotides were synthesized, annealed and inserted to the pcDNA6.2-GW/EmGFP-miR vector. The pcDNA6.2/EmGFP-miR-neg control plasmid (neg-miRNA) was used as a negative control.

### Cell transfection

Neurons cultured for 7 days in vitro or HEK293 cells were transfected with plasmids pEGFP-C3-rBNIP3, pEGFP-C3, BNIP3-miRNA and neg-miRNA as we described previously [11] using Lipofectamine 2000 (Invitrogen) according to manufacturer's recommendations.

### Statistical analysis

All data are given as means ± SE if not indicated otherwise. One-way ANOVA was used to test for overall statistical significance. A difference was considered significant at *P *< 0.05.

## Authors' contributions

TMG, XHZ, JMK and MC designed the study. STZ and SJL cultured the cells and performed OGD. MC generated the miRNA plasmids. STZ carried out cell transfection. STZ, MHZ and BXL contributed to the western blot analysis and immunocytochemistry. MC and STZ drafted the manuscript. TMG, XHZ, MD and JCB finalized the manuscript. All authors have read and approved the final manuscript.
